# How do former medical and nursing undergraduates describe their learning on an interprofessional training Ward 12–18 months later? – A retrospective qualitative analysis

**DOI:** 10.1186/s12909-023-04212-5

**Published:** 2023-04-21

**Authors:** Johanna Mink, Bianka Zurek, Burkhard Götsch, André L. Mihaljevic, Anika Mitzkat, Birgit Trierweiler-Hauke, Cornelia Mahler

**Affiliations:** 1 Department of General Practice and Health Services Research, University Hospital Heidelberg, Heidelberg, Germany; 2 Faculty of Social Sciences, Institute of Sociology, Goethe-University Frankfurt am Main, Frankfurt, Germany; 3 Nursing School, Academy of Health Professions Heidelberg, Heidelberg, Germany; 4 Department of General and Visceral Surgery, University Hospital Ulm, Ulm, Germany; 5 Department of General, Visceral and Transplantation Surgery, University Hospital Heidelberg, Heidelberg, Germany; 6 Department of Nursing Science, University Tübingen, Tübingen, Germany

**Keywords:** Interprofessional Education, Nursing education, Medical Education, Interprofessional collaboration, Professional competence

## Abstract

**Background:**

Interprofessional training wards (IPTWs) seem to deliver good results in terms of development of interprofessional competencies. However, evidence of long-term effects of these training wards on learners’ competency development is lacking and little is known about retrospective evaluation of IPTWs. Therefore, this study aimed to explore the retrospective evaluation of competency development and interprofessional collaboration of former undergraduates 12 or more months after a placement on an IPTW.

**Methods:**

Eight follow-up interviews were conducted with four nursing and four medical professionals 12–18 months after they had finished a placement on an ITPW throughout their vocational training. Interviews were translated verbatim and analysed deductively and inductively based on qualitative content analysis.

**Results:**

The qualitative content analyses deductively identified two main categories regarding the research question, namely the uniqueness of the programme and interprofessional competencies developed by the Interprofessional Education Collaborative. Sub categories were identified inductively, representing the perceived competency development and the learning opportunities on the IPTW as compared to other clinical placements throughout vocational training and in transition to practice. Interviewees seemed to have developed competencies that are important for interprofessional collaboration such as communication, roles and responsibilities, as well as competencies in patient care and management. Considered beneficial for learning were the opportunity to work self-responsibly and the interprofessional collaboration on the IPTW, both of which were neither possible in almost any other placement nor in transition to practice.

**Conclusion:**

Findings show that IPTWs can be sufficient in competency development and role clarification and are perceived positively by learners, but structures in clinical practice can impede sustaining competency development and efficient interprofessional collaboration.

## Introduction

Interprofessional education (IPE) enabling learners of different health care professions “*to learn about, from and with each other”* [1] is gaining importance within the education of health care professionals. IPE can result in improved interprofessional collaborative practice (IPCP), which “*occurs when multiple health workers from different professional backgrounds provide comprehensive services by working with patients, their families, carers and communities to deliver the highest quality of care across settings.*” [1]. IPCP can have positive effects on patient care and staff satisfaction [2] and IPE in real clinical settings with real patients has proven to be effective in the development of IPCP [3]. This can be provided on student-led clinics or interprofessional training wards, which are in-patient wards in clinical settings where undergraduates of various health care professions are responsible for collaborative patient care during their vocational training [3]. Interprofessional training wards (IPTWs) have shown to exhibit high quality of patient care [4, 5], improve patient satisfaction [6] and are considered to be an effective educational method to develop interprofessional competencies among health professionals [3, 7]. Pre- post-evaluations of the first IPTWs in Germany, with implementation as of 2017 [8, 9], show that learners’ competencies in teamwork and collaboration, as well as attitudes towards interprofessional learning and interprofessional interaction improved significantly at the end of a placement on an IPTW [10–12]. Existing evidence related to the long-term value of IPE indicates that this improvement is not sustained: Statistically significant decreases of learners’ attitudes towards interprofessional learning, interaction and teamwork have been observed in follow-up measures after graduation and while working in clinical practice compared to measures directly after IPE [12, 13]. A reason for the decrease of effects is that interprofessional collaboration (IPC) in everyday routine clinical practice differs from what is taught during IPE, as has been demonstrated in the field of oral care education [14]. Further hindering factors for IPCP in clinical practice are less time or lack of collegial support [15] as well as existing hierarchies in clinical practice [16]. In terms of attitudinal and behavioural change it has been demonstrated that a majority of individuals have implemented certain commitments regarding IPC in their clinical practice two months after a simulation-based IPE training [15]. Still, there is a shortage of studies analysing the long-term effects and outcomes of clinical interprofessional training [17, 18]. The first long-term evaluations of IPTWs in Germany show positive effects on medical and nursing trainees’ interprofessional competencies 3 to 34 months after a placement on an IPTW, even though the effects decrease compared to directly after the placement [11, 12]. The aim of this study was therefore to gain insight in the retrospective evaluation of perceived competency development and interprofessional collaboration 12 or more months after a four-weeks-placement on an IPTW compared to other clinical placements during training and clinical practice after graduation by the participating former nursing and medical undergraduates. Interprofessional competencies are analysed according to the framework of the Interprofessional Education Collaborative (IPEC) distinguishing the following competency domains:

- Values and Ethics in Interprofessional Practice (VE).
- Roles and Responsibilities (RR).
- Interprofessional Communication (CC).
- Teams and Teamwork (TT) [19].

## Methods

Convenience sampling of 60 participants that completed the four-weeks placement on the Heidelberg Interprofessional Training Ward (Heidelberger interprofessionelle Ausbildungsstation, HIPSTA) [8] during 2017 and 2018 was undertaken by the author (JM) who recruited participants via mail, if contact data were available. During four-weeks placements on HIPSTA four nursing undergraduates in their last year of vocational training (third year) and four medical undergraduates in their clinical year after 5 years of studies (sixth year) were responsible for patient care on a surgical ward, supported by selected medical and nursing facilitators from the respective ward [8]. The participants invited for this study were former medical and nursing undergraduates who were part of the evaluation of the HIPSTA 2017–2019 [9]. Accordingly, the author, who worked as a research fellow in the project with a professional background in gerontology, didactics and interprofessional education and experiences in qualitative research, was already known to the participants as one of the researchers responsible for the evaluation of the HIPSTA. The invitations for participation were sent out twice to the first seven cohorts (n = 47 persons) and once to participants of the cohorts eight and nine (n = 13 persons), each 12 to 18 months after the end of the individual’s HIPSTA-placement. A total of 8 persons communicated interest in participation by replying to the e-mail invitation. A semi-structured interview guide was developed by the author (JM) in iterative collaboration with another researcher (CM) to facilitate generating narratives from the interviewees. Main aspects of this guide were the general experience of the placement on HIPSTA, the clinical placements afterwards, transition to practice after graduation, interprofessional collaboration and competency development. The main questions of the guide can be found in Table 1.

**Table 1:** Main questions of the interview-guideline

Topic	Question
1. Getting started	How was the placement on HIPSTA for you? What were decisive experiences?
2. Post-HIPSTA training	Was the placement on HIPSTA the last one before your examination? *If no*: How were the consecutive placements within your medical / nursing training?
3. Transition to practice	How did you perceive the entrance into working life? • Main challenges? • What was easy?
4. Interprofessionality	On HIPSTA you worked together with learners of another profession – how did you perceive this collaboration? How do you evaluate the interprofessional collaboration in the clinical practice?
5. Competency development	HIPSTA is a special learning format, in which you are responsible for patient care together with learners of another profession. How do you evaluate this type of education throughout training? How do you evaluate the sustainability of the competencies you developed during your placement on HIPSTA? To what extent could you use the experiences you made on HIPSTA in your following placements? How has the placement on HIPSTA changed your perception of your own profession?
6. Closure	Is there anything else, you would like to mention?

All interviews were conducted by JM, audio-recorded, and transcribed verbatim. For data analysis, the written transcripts were pseudonymised based on the professional background of the participants (P for physicians and N for nurses) and randomly assigned numbers from one to four for each profession. The qualitative content analysis [20] deductively identified categories related to the research question and along the topics of the semi-structured interview guide (see Table 1). In addition, for deductive coding of the subcategory „IP competencies“, the framework for interprofessional competencies developed by the Interprofessional Education Collaborative [19] was used. Within the main categories, further subcategories were identified inductively. Coding was performed by JM using MAXQDA Software [21]. In order to provide intersubjective reliability, the author (JM) discussed the process and findings of the analysis with two researchers with professional background in sociology (BZ, B.A.) and nursing sciences and interprofessional health care (CM, Prof.) and if necessary adapted them until agreement was reached. Quotes were translated to English for presentation in this manuscript.

## Results

### Sample

Eight interviews were conducted with four female nursing professionals (N1, N2, N3, N4), and four medical professionals (P1, P2, P3, P4), of whom two were male and two female, 12 to 18 months after their placement on the IPTW. All of the participants had further placements after their placement on HIPSTA mainly in settings like internal medicine or intensive care. At the time of the interview, the former nursing undergraduates were all working as registered nurses in inpatient care (N1, N4), intensive care (N2), and emergency care (N3). Two of the former medical undergraduates were working as physicians with diagnostic and therapeutic specializations (P1, P3), one was undertaking a medical internship abroad for further training after graduation (P4), and one had changed the field of practice, which had already been envisioned before the medical studies and which also includes personal contact, exchange and consultation (P2). The interviews lasted on average 26 min (from 15 to 30 min) and were conducted via telephone (n = 7) and face-to-face (n = 1) in the office of the researcher (JM). After eight interviews were conducted with an equal number of nursing and medical professionals, saturation was reached in so far as that substantial codes were identified in advance (deductive codes) or within the analysis of the first two interviews (inductive codes). The stable category system comprised mainly concrete categories based on an existing framework, earlier evaluation and the research interest [22]. The group of participants was considered homogenous in terms of experience on HIPSTA and level of interprofessional education and adequate to provide a first impression of nursing and medical participants’ experiences [23].

### Categories

Two main categories and six sub-categories were clustered deductively. An overview of the categories is given in Table 2.

**Table 2:** Main- and sub-categories identified within the qualitative content analysis

Main-Categories	Sub-Categories
Uniqueness of HIPSTA	Learning by doing
	Working and learning together in an interprofessional environment
	Clinical nurse/physician facilitators
	Comparison to other clinical placements during training and in clinical practice
Competency development	IP competencies
	Organisation and management
	Patient care
	Confidence and self-efficacy

### Uniqueness of HIPSTA

Within this category, statements are clustered in four subcategories that deal with determinants for learning on HIPSTA and the comparison of HIPSTA with other clinical placements throughout professional training.

#### Learning by doing

One nursing and four medical participants described that taking over responsibility for the whole process of patient care on HIPSTA, from admission to discharge and post hospital care, was beneficial for developing competencies in medical and nursing care, as well as in care organisation and management. Carrying out tasks self-responsibly was described as helpful for a better overview of care processes and roles and responsibilities of the professions within health care. It was perceived to initiate reflection on patient care, active decision-making and foster self-directed knowledge acquisition.

*“… and since you didn’t just help there, but were primarily responsible for it, I at least engaged much more intensively and worked on it much more intensively than I usually did in the practical year and medically, I also learned an insane amount there.“* (P1).

Being able to learn in a secure environment where mistakes could be made and things tried out was described as helpful for professional development.

#### Working and learning together in an interprofessional environment

Learning from and with each other was mentioned as beneficial for the development of (inter)professional competencies.

“*That you just learn from each other on a daily basis, I thought that was nice. Everyone knows a little bit more about one area and then, when you share it with each other, it’s more productive for everyone.*“ (P4).

Participants explained how they practiced specific skills together. Some of them mentioned the benefits they still experienced due to these skills within their daily practice.

„…s*o we also did things like measuring blood pressure in our ward room, or the doctors or the medical undergraduates measured our blood pressure, and we inserted a needle or took blood from the medical undergraduates, so we exchanged tasks a little bit*.“ (N2).

Shared working hours and breaks on HIPSTA were described as intensifying mutual contact within the heterogenous team and thus improving IPC and socialisation. Learning together and working together towards common goals on HIPSTA was defined as positive experience that could not be replicated in clinical practice afterwards.

#### Clinical nurse/physician facilitators

Participants also mentioned the nursing and medical facilitators as supportive by being role models, professional experts and feedback-givers.

“…*and I thought it was great that we were closely supervised by the nursing staff. And I had the impression that the nursing and the medical leaders communicated well with each other, which of course had an effect on our cooperation.*“ (P4).

### Comparison to other clinical placements during training and in clinical practice

#### Clinical placements during training

The most important difference between the placement on HIPSTA and other placements described by medical participants was that they normally were not allowed to take over patient responsibility.

„…*the other rotations were just the way it usually is, so you don’t have your own patients, you run around and draw blood, you don’t know any patient stories, you don’t learn much, you do whatever work comes up, you don’t have any contact with attendings, you don’t get taught anything, just because, yeah, well, no comparison.*.“ (P2).

Nursing and medical participants reported to have learned more on HIPSTA than during any other clinical placement. Working and learning self-directedly or even asking questions during ward rounds was claimed as not possible in most other clinical placements, due to lack of time or strict hierarchies.

“*So, when I then started the consecutive placement, I had to slow down a bit […], because the structure of the ward rounds there was so strict that the nurses had nothing to say, they just walked along and held the files, and that was extremely difficult for me, because it was always itching in my fingers: I would like to get involved now, but I can’t, because it’s just not welcome there*.“ (N1).

#### Clinical practice in general

Participants portrayed differences of IPC depending on clinical setting and discipline and explained that both in clinical practice and training, barriers between the professions had been observed which were not perceived on HIPSTA.

*“… but now, looking at professional aspects, I thought it [placement on HIPSTA] went well. So you were not so isolated as in normal routine, where you have a strict separation between physicians and nurses everywhere, but there [on HIPSTA] it was rather, yes, a team, sometimes more sometimes less, but rather better than in clinical routine.”* (N2).

Compared to the situation of mutual acceptance on HIPSTA, existing hierarchies in everyday clinical practice were described.

*„…and then you also have a bit more acceptance, because sometimes it is the case that some physicians put themselves above the nurses.“* (N4).

A medical participant described IPC in certain clinical settings as “clumsy” and explained that with existing hierarchies and lacking direct communication.

„…*I think that they, especially because – in my opinion – from the physicians‘ perspective towards the nurses, that they, I had the impression, they thought that nursing was inferior for certain things*…” (P4).

Malfunctioning communication, hierarchies, stereotypes and barriers between the professions in clinical practice were regarded as threats for patient safety. The lack of opportunities for interaction between professions was mentioned as a reason for bad IPC and “resentments” (P1) between the professions.

Two nursing professionals, who worked in intensive and emergency care settings (N2 and N3) described their current work very positive regarding IPC. They commended structures in their clinical working environment that enabled exchange, reflexive rounds within the multiprofessional team, continuing contact between the professions throughout the shifts and mutual support.

„…f*or example, when we had a difficult case or it went somehow chaotic or complicated, then we often reflect together. And I think this is very nice. Right. That we then give each other a critique to take along and see what we could do better next time. […] That it functions somehow. And, of course, that it thereby is beneficial for the patient.”* (N3).

#### Competency development

Four main areas of described competencies were identified in the analysis, namely interprofessional (deductively), organisational, patient care, confidence and self-efficacy (inductively). In general, all participants described a profound competency development throughout their placement on HIPSTA.

#### Interprofessional competencies

Participants’ statements were clustered deductively according to the four competency domains of the IPEC framework [19].

### Values and ethics in interprofessional practice (VE)

A nursing participant described an increased understanding and valuing of the medical profession. A medical participant described a better understanding of the aspects that need to be considered in patient care and how this change in attitude and knowledge helped to plan and conduct patient care in further clinical practice.

„*And that you perceive the patients more as persons than as someone with a disease. That you really care about them. Not only acutely, during the stay, but and above all also for the life afterwards. One understands that the disease does not end at the main entrance or the main exit*.“ (P4).

### Roles and responsibilities (RR)

A nursing and three medical participants mentioned the development of competencies in dealing with roles and responsibilities. Advantages of the other profession’s expertise to broaden the own competencies and a better understanding of the other profession’s tasks were described. This understanding and interprofessional exchange was described as beneficial for collaboration in their current clinical practice. Two participants described an adaptation of their own way of working in order to meet the other profession’s needs and hence improve collaboration.

“…*that you also had to somehow distribute the tasks and also figure out, this is what I have to do in my service and that can perhaps be done by the medical undergraduate. […] And I think that this has also brought me a bit further, that I have thought about what I have to do and could somehow coordinate myself much better with my schedule and my work plans after the placement.*“ (N4).

### Interprofessional communication (CC)

Except for one medical participant all others described an acquirement of competencies in interprofessional communication during their placement on HIPSTA, which was still perceived as beneficial in current practice. Mentioned were the improved structure of communication due to tools or self-developed rules for communication and the competence to adapt communication style to the knowledge and needs of recipients when passing over information or participating in a discussion.

„…*and also through this whole SBAR [Situation, Background, Assessment, Recommendation] model to concretize things relatively quickly and concretely on the phone and to say, I need your help now and this is the problem. Right. I have simply profited extremely from it.*“ (N1).

Especially the medical participants reported to have improved competencies in talking with and listening to patients.

„*…filtering out unimportant information has become easier for me. That you understand the patients more quickly and understand more quickly what kind of problem they have, why they are there and what needs to be done*.“ (P3).

The importance of open exchange and shared knowledge within patient care was mentioned and the own contribution to that was reflected.

### Teams and teamwork

Five participants expressed a general increase of teamworking skills or “team competency”. The three nursing and two medical participants described that integrating themselves into new working teams in transition to practice was perceived as easy.

„…*HIPSTA was definitely not detrimental to my team-competencies; I rather think it was positive. Especially, of course, in the context of cooperation with nursing. There HIPSTA, of course, was enormously positive*.“ (P1).

One nursing participant described the supportive structures at the current workplace setting including open-minded colleagues and structured feedback sessions.

*„I don’t know now whether it’s necessarily because of HIPSTA or also because of our doctors. We reflect a lot after [collaborative] actions. For example, when we had a difficult case or it was somehow chaotic or complicated, then we often reflect together.” (N3)*.

One nurse mentioned that competencies in terms of conflict resolution and a better ability to openly communicate critique were developed during the placement on HIPSTA.

#### Organisation and management

One nursing and two medical participants reported a better understanding of holistic patient care and health care organisation, as well as ward management competencies.

„*Then, in general, how to run a ward. So, in fact you only have one room. But you can multiply that by ten or whatever. (laughs) Right. So, the whole organization. You have to fulfil so many non-medical tasks on the ward. Um, I was already aware of that before. But how it really works and what has priority now and when do I have to call the senior physician. You don’t learn that in your studies*.“ (P3).

#### Patient care

Statements regarding post-operative care on a surgical ward were clustered inductively. Competencies in wound care, management of drainages, pain therapy, nutrition, mobilisation, placing an i.v. access or hygiene were described. One medical participant specified that these competencies were developed on HISPTA only and another one explained how acquired competencies were beneficial for further practice in other disciplines.

„…*I learned how to properly care for pancreatic cancer patients, how to follow up splenectomized patients, I dealt with acute abdomen, I dealt with hypertensive crises, actually also internal medicine emergencies. Then, I think there was a patient where we had to probe wounds, I learned that often when patients’ blood pressure shoots up, it’s not because of the blood pressure itself, it’s because they don’t have sufficient pain management, setting pain management, discussing rounds correctly with people. Right. And dressing changes.*” (P4).

A deeper knowledge about diseases and about the interpretation of diagnostics, lab results and x-rays was described.

„…*So especially on HIPTSA I also learned to look at the lab results, too and to pay attention to the lab of my patient on my own, and this is what I have to do now as well…*“ (N4).

Some participants mentioned that these clinical competencies were less sustained than interprofessional competencies due to differences in clinical settings they worked in. For example, if post-surgical treatment competencies were not needed after their HIPSTA placement, these were not consolidated in diagnostic healthcare settings. On the other hand, participants also described that their vocational education can only enable development of basic or generic competencies which often needed further development within transition to practice in the wide range of healthcare settings. Accordingly, for health care competencies also gathering of practical experience was described as an important influencing factor.

#### Confidence and self-efficacy

Participants described sustained self-confidence, self-efficacy and trust in the own competencies due to their experiences on HIPSTA.

„*And I was indeed extremely nervous before. But then in the course of it, um, a kind of self-confidence built up and somewhat the faith in yourself, I’d say, has strengthened*.“ (P3).

Own demeanour in interaction with patients and other professionals was perceived as strengthened and their own profession represented with more confidence, which was also described as useful in transition to practice.

„…*and in addition to that, the independent part was just, yes, that you learn a little bit more to listen to yourself, to stand up for yourself, to express your own opinion.*“ (N4).

All medical professionals described the experience of self-efficacy and meaning in their own work. It seemed to have become clearer what being a physician means.

„*That was this positive self-affirmation that you could actively do something, and that this then also really has direct effects for patients and you have the feeling that you are doing something meaningful the whole day and not just taking blood or writing out some doctor’s letters*.“ (P1).

## Discussion

### Summary

Findings of this study show that 12 to 18 months after the placement on the IPTW, participants described the positive impact of the placement on their development of interprofessional competencies, particularly in the domain *interprofessional communication*, but also of professional competencies in terms of diagnostics and treatment, as well as organisation of care processes. Participants described increased self-confidence, which was perceived as beneficial for further placements and transition into professional practice. Structures of the IPTW were experienced as beneficial to competency development, notably the opportunity to take over responsibility and the interprofessional setting. IPC and IPE on the IPTW was described as more positive compared to other clinical placements within vocational training and clinical practice.

### Learning and growing on HIPSTA

The students experienced the interprofessional setting as positively influencing their development of (inter)professional competencies by learning from and with each other. Intense contact and mutual trust in the group of learners created a safe environment, in which learning was facilitated and the interaction with each other was practiced. Most notably, competency development was perceived in the domain of interprofessional communication. This is complementing the results of Mahler et al. 2018, who detected difficulties in this competency domain in a qualitative study of IPE at earlier stages in professional training [24]. One explanation for this difference can be the practical context. Interprofessional learning in a simulation-based or practical setting has been shown to be the most effective in terms of interprofessional competency development [25]. In IPE-settings with real or simulated patients, learners were able to learn about roles, improved their confidence in communicating and developed an appreciation of IPE as positive for patient care [26]. Learning on HIPSTA occurred self-organised, in interaction with others and also informal, which can be beneficial for competency development. These findings are in line with the results of the longitudinal quantitative analysis of the learners’ competency development on HIPSTA, which showed significant improvement of self-perceived competencies in terms of communication, teamwork, interprofessional learning, and cooperation three months after placement as compared to before placement on HIPSTA [12].

Findings of this present study also indicate that self-confidence was strengthened, which was perceived as beneficial for transition to practice and fulfilling the role of a nurse or a physician. Literature shows that role models, experiential learning and reflective processes can foster socialization and thus professional identity formation [27, 28]. These aspects seem to be fulfilled on HIPSTA, as the interprofessional setting initiates reflection on roles and responsibilities, the facilitators serve as role models and mentors and the learners can make their experiences via learning by doing. Studies have shown that working self-responsibly and „trying on“ the professional role while supervised by facilitators can be perceived as beneficial for learning and professional development by medical students [29–31].

Even though the continuous interprofessional contact on IPTWs has been criticised as artificial, and variations with medical undergraduates leaving the IPTW for several hours have shown positive impact on role clarification and communication [32], it has also been shown that a sense of belonging to a health care team on an IPTW is appreciated by the undergraduates [33]. In the reconstructive analysis of socialization processes on HIPSTA it became obvious that learners experience a sense of belonging to the group of undergraduates during their placement [34]. In this study, the retrospective appreciation of shared working hours and breaks supports the importance of relatedness and intense contact, which can also result in informal learning processes.

### Challenges and chances in practice

Participants described restrictions in clinical placements after HIPSTA, where working on an equal level and self-responsibility were not facilitated. They named hierarchical structures and a lack of time as hindering factors for self-directed supervised learning and efficient IPC. Such organizational and inter-individual factors have been described most often as determinants of IPC in primary care [35]. For successful implementation of IPC into the workplace setting, relational, processual, contextual, and organisational factors need to be considered [36, 37], instead of assigning the task of implementation of teamwork to the newly graduated professionals [17]. In line with this, the two nursing professionals in this study who evaluated IPC in their clinical setting positively mentioned respective structures as facilitating factors. Furthermore, their respective workplace settings (intensive and emergency care) are influenced by often unexpected and urgent events that require complex team tasks and thus close interprofessional teamwork, which is characterized by a sense of belonging to the interprofessional team and mutual dependence and support [37]. IPCP on HIPSTA was described very positively as close and interdependent in a reconstructive analysis of learners’ identity development directly after the placement [34] and was still remembered vividly by the participants in this study after 12–18 months. Still, the abovementioned reconstructive analysis demonstrated that a four-weeks placement on an IPTW might be not sufficient for forming an interprofessional identity, since the sense of belonging the undergraduates described is rather related to the group of learners than to the group of health care professionals [34]. Continuing IPCP and close contact to other professionals would be needed also within transition to practice in order to strengthen the interprofessional identity. However, it became obvious that even though the participants benefitted from the placement on HIPSTA, most of them experienced disappointment in consecutive clinical practice. This confirms findings of a prior quantitative analysis of competency development on HIPSTA where participants scored their perception of collaboration in clinical practice three months after the placement on HIPSTA not significantly better than before [12]. In order to prepare future professionals to deal with the IPE – IPCP – gap, more emphasis should be put on (inter-)professional identity formation, open communication about stereotypes and hierarchies and competency development in the field of change management within training. In addition to these relational and processual factors, the leadership, the system including politics, community and health insurances need to actively demand and enable interprofessional collaboration in order to deliver a solid basis for a sustaining interprofessional collaborative practice [39].

### Limitations

The aim of this study was to explore perceptions of HIPSTA group members. The sample consisted of eight former undergraduates from five out of nine HIPSTA cohorts. It cannot be excluded that a larger sample representing the HIPSTA group might have allowed for additional or different findings. However, we consider the participant’s perception of their HIPSTA-experience a strong and relevant account of the HIPSTA contribution to interprofessional learning. This account can provide the base for larger studies investigating similar student experiences and inform their considerations of interprofessional competency scales. To what degree participants can integrate interprofessional competencies in their daily practice is not clear, as all identified related aspects were self-reported. However, they all seemed to have a positive attitude towards IPC. In order to reach intersubjective reliability, coding was performed with an independent researcher who was not involved in HIPSTA implementation or the research to date (BZ). Future use of a validated interprofessional competency scale over time could strengthen the results in further studies. Using the interprofessional competencies outlined by the IPEC as structuring framework might have narrowed the scope of detected competency development during analysis. Still, it enabled structuring and comparing individual descriptions of self-assessed competencies.

## Conclusion

Even 12 to 18 months after placement on HIPSTA, former participants saw the benefits of their placement for future clinical practice after transition to practice. The combination of learning by doing, facilitation and interprofessional learning on an IPTW was regarded as beneficial for competency development and a further step toward professional identity formation of participants. Most of the interviewees perceived less interprofessional collaboration and communication in their consecutive placements and experiences in clinical practice than on HIPSTA. This shows the relevance of IPTW in order to foster IPC, the need to embed clinical and authentic patient care-based interprofessional education throughout the curricula of all health professionals, and the importance of strengthening IPC in health care.

## Data Availability

The datasets generated and analysed during this study are not publicly available due to data protection regulations, but are available from the corresponding author on reasonable request.
